# Spatial and Seasonal Dynamics of Water Environmental Capacity in Mountainous Rivers of the Southeastern Coast, China

**DOI:** 10.3390/ijerph15010099

**Published:** 2018-01-09

**Authors:** Qiankun Liu, Jingang Jiang, Changwei Jing, Jiaguo Qi

**Affiliations:** 1 Ocean Colleage, Zhejiang University, Zhoushan 310058, China; lqk19890627@163.com (Q.L.); gangzg@163.com (J.J.); 2 School of Science, Hangzhou Normal University, Hangzhou 310018, China; 3 Center for Global Change and Earth Observations, Michigan State University, East Lansing, MI 48824, USA

**Keywords:** water environmental capacity, one-dimensional WEC model, pollution load distribution, water quality, WEC spatial and seasonal patterns

## Abstract

The south-east littoral is one of the most populous and developed regions in China suffering from serious water pollution problems, and the Xian-Jiang Basin in the mid of this region is among the most polluted watersheds. Critical information is needed but lacking for improved pollution control and water quality assessment, among which water environmental capacity (WEC) is the most important variable but is difficult to calculate. In this study, a one-dimensional water quality model combined with a matrix calculation algorithm was first developed and calibrated with in-situ observations in the Xian-Jiang basin. Then, the model was applied to analyze the spatial and temporal patterns of WEC of the entire basin. The results indicated that, in 2015, the total pollutant discharges into the river reached 6719.68 *t*/yr, 488.12 *t*/yr, and 128.57 *t*/yr for COD, NH_3_-N and TP, respectively. The spatial pattern suggested a strong correlation between these water contaminants and industrial enterprises, residential areas, and land-use types in the basin. Furthermore, it was noticed that there was a significant seasonal pattern in WEC that the dry season pollution is much greater than that in the plum season, while that in the typhoon season appears to be the weakest among all seasons. The WEC differed significantly among the 24 sub-basins during the dry season but varied to a smaller extent in other seasons, suggesting differential complex spatial-temporal dependency of the WEC.

## 1. Introduction

Coastal regions, where about 70% of the population and industrial capitals worldwide, are full of well-developed metropolitan economic corridors, leading to serious water shortages and environment deteriorations, due to rapid urbanization and industrialization [1,2]. In recent years, total water pollution control as a policyinstrument has been adopted by environmental protection agencies in China to meet the national water quality standards [3] and to ensure drinking water safety. As the premise and foundation of water pollution control, knowledge of water environmental capacity (WEC) plays a critical role [4,5,6]. Like total maximum daily load (TMDL), the concept of WEC refers to the total maximum load of pollutants that a waterbody can accommodate without significantly affecting its environmental function and water quality standards set forth by environmental agencies [7,8,9]. Coastal climatological and orographic characteristics where mountainous land and plain crisscross (influenced by a marine monsoon climate), lead to an obvious dissimilarity in rain-runoff in space and time [10,11] that is likely to result in different WEC. However, existing WEC modeling is insufficient and few models consider spatial heterogeneity and seasonality. Therefore, improved modeling efforts on WEC that specifically consider spatial heterogeneity, seasonality, and hydrological processes are needed in order to better manage environmental pollution and water quality control.

Many models, some complex and some simpler, have been developed over the past decades to estimate water quality in rivers [12,13], including the S-P models [14], Water Quality Analysis Simulation Program (WASP) [15], STREAM [16], and QUAL2K models [17]. Complex models capture physical and chemical processes but require many water quality parameters and, thus, are difficult to apply in practice given the lack of long-term data availability for most watersheds in China [9]. Lindenschmidt indicated that complex models were not necessarily the most useful ones, especially for mountainous streams, where some processes involving algae and sedimentation can be negligible due to shallow and fast-moving water, and few river impoundments [18,19]. Some models are specifically parameterized with empirical data and may include parameters that have never been previously measured or reported. Therefore, use of complicated models for water quality simulation might not be as practical as purported [20]. Given these circumstances, the utilization of simple and suitable water quality models for mountainous rivers has become a preferred option.

In addition to model options, the way spatial resolution or grid size model is run has a direct effect on simulation accuracy. The finer the grid is, the better pollutant transform can be modeled; thus, the more detailed WEC distributions. The spatial grid size is particularly important for river basins of complicated topography and landscape in mountainous regions with complicated channel characteristics. This requires finer spatial resolution or grid to capture the complex river processes. Most one-dimensional models are run with 500 m or coarser resolutions, due to computation limitations and time [16,19]. Few models have been used at a resolution higher than 100 m, to our knowledge, to simulate WEC across space-time in large river systems. Therefore, to capture the dynamics of spatially heterogeneous river basins, especially those basins of limited in-situ data, it is desirable to have a simple model capable of fast and fine grid simulations.

In this paper, a model to capture the pollutant water quality responses was developed based on the Manning equation and the Streeter–Phelps integration, combined with a matrix calculation algorithm that allows simultaneous simulations of contaminant concentrations in all grid cells. The primary objective was to develop a simple, process-based, model that can practically characterize and analyze the temporal and spatial distribution of WEC at basin scale, thus generating cirtical information of the Xian-Jiang basin for informed environment risk management.

## 2. Study Area and Methods

### 2.1. Study Area

**Figure 1:**
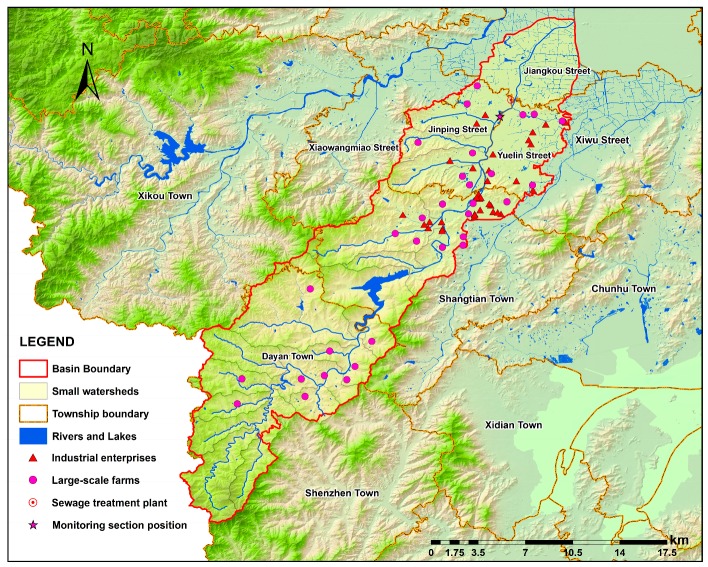
The geographic distribution of rivers and pollution sources situates in Xian-Jiang Basin.

The study area (Xian-Jiang Basin) is located in Ningbo, Zhejiang Province in the southeast of China, with a drainage area of 306.70 km^2^ of 15 main rivers (Figure 1). The area has a subtropical monsoon climate that encompasses a dry season from December to January and a plum rain season from May to June. The time period from July to September is the typhoon season, when rains are mixed with typhoon storms [21]. The basin’s terrain transitions from mountain topography in the west to the gentle plain in the coastal east. The basin provides an important source of drinking water for Ningbo City.

In spite of its importance as Ningbo’s primary drinking water source, the basin is among the most industrialized cities in China, experiencing serious freshwater pollution problems over the past three decades that threatens water environment and drinking water security [22,23]. The primary water pollutants found in the water are chemical oxygen demand (COD), NH_3_-N, and total phosphorus (TP). The poor water quality was attributed to the industrial plants, residential sewage, agricultural run-off, large-scale livestock culture, and wastewater treatment plant discharges (Figure 1) within the basin.

### 2.2. Hydrological Characteristics of Xian-Jiang Basin

**Figure 2:**
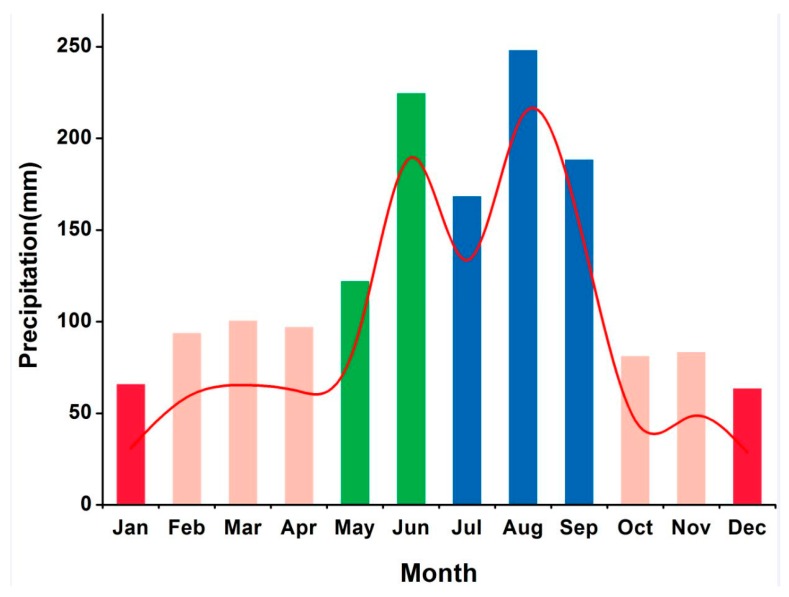
The 2001–2015 monthly mean precipitation in the Xian-Jiang Basin.

The average monthly rainfall over the last 15 years (2000–2014) recorded at the three rain gauge stations in the Hong-Jia-Ta catchment with the area of 151 km^2^ near the basin shows a bimodal pattern (Figure 2). The monthly average precipitation over the last 15 years ranged from 50 mm in the winter season to 250 mm in the summer season. The heaviest rainfall (more than 248 mm) occurred in June and August; July and September also received high rainfall, while the minimum rainfall occurred in December with only 63.53 mm. Rainfall intensity in the dry season, plum rain and typhoon seasons were 129.42 mm, 346.76 mm, and 604.99 mm respectively, accounting for 8.42%, 22.56%, and 39.36% of the annual total precipitation.

**Figure 3:**
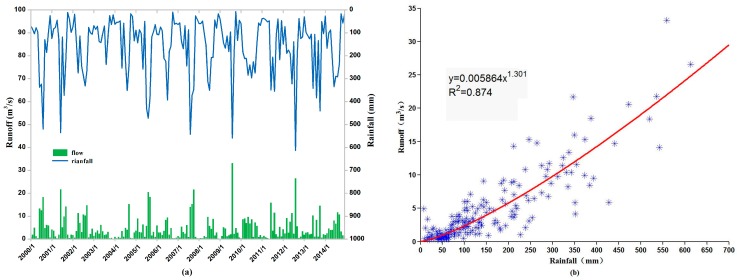
The correlation between rainfall and runoff: (**a**) Monthly change of rainfall and runoff during the last fifteen years; (**b**) Fitted equation of precipitation and runoff *y* = 0.005864*x*^1.301^, *R*^2^ = 0.874.

Long-term hydrological observations at Hong-Jia-Ta hydrological station in Ningbo City were used as the monthly run-off data (2000–2014) in the Hong-Jia-Ta catchment, as it is the only station closest to the basin. Both rainfall and runoff appear to have a similar pattern (Figure 3a), and there is a high correlation that can be fit with an exponential function (Figure 3b) [24]. The explanation power or correlation coefficient is 0.87, which suggests that runoff can be approximated by its rainfall patterns. The streamflow in the Xian-Jiang basin and its sub-basins can, therefore, be estimated using precipitation data (2015) from Feng-Hua station within the basin through the hydrologic analogy method (Equation (1)) [25].

(1) $$Y_{i}=\frac{k_{i}}{K}\left(0.005864x^{1.301}\right)$$

where $Y_{i}$ is the ith sub-basin’s monthly runoff (m^3^/s); $k_{i}$ is the area of ith sub-catchment (km^2^); K is the area of Hong-Jia-Ta catchment; x is the monthly precipitation of the basin in 2015 (mm).

### 2.3. Calculation and Allocation of Pollution Loads

The pollution sources in the basin were identified mostly from agricultural non-point sources, large-scale breeding farms, domestic sewage, industrial enterprises, and a wastewater treatment plant. The load from the first two sources was computed by using Johnes model (Equation (2)) [26]. The rate loss of various agricultural non-point sources loads (paddy field, dry land, economic forest, and garden) was determined based on previous studies citing analogy basins [27,28,29]. For the domestic sewage, there are primarily three mechanisms to discharge pollutants into rivers, according to a field survey and a review of previous studies: (1) sewage pipes divert effluence into rivers and streams after a treatment by sewage disposal facilities in villages; (2) the effluent is discharged directly into rivers and streams after a simple treatment by septic tanks; and (3) all others are all discharged into a sewage network and eventually enter sewage treatment plants before being discharged into streams and rivers.

The pollution loads for each of the three were calculated in this study. The pollution loads of 36 key industrial enterprises and wastewater treatment plants in the basin were obtained from the environmental statistical data of Ningbo city in 2015. As for pollution loads allocation into rivers, the pollution load from plants (including the wastewater treatment plant) discharged directly to the reaches where their outlets lay.

**Figure 4:**
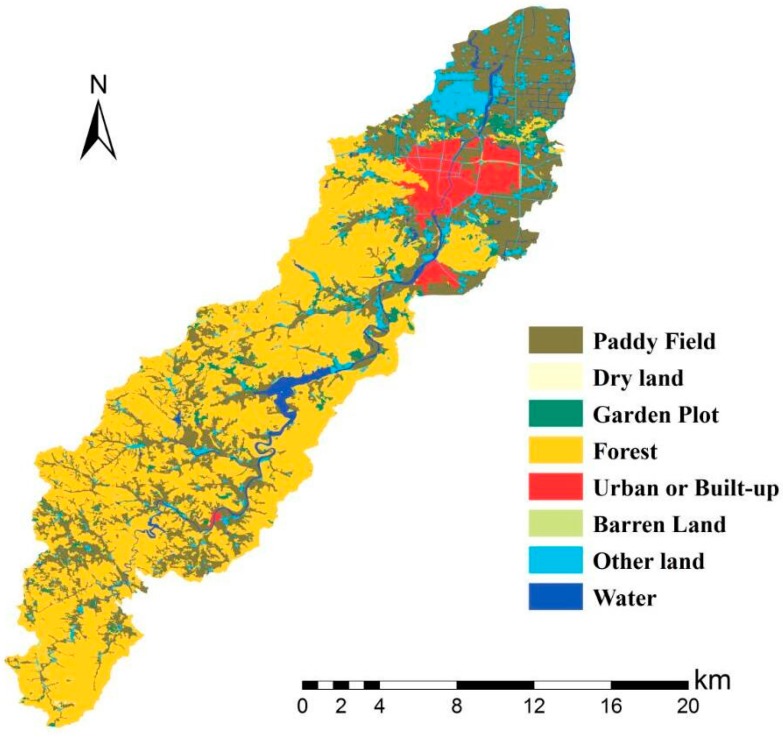
The land use and land cover of Xian-Jiang basin in 2015.

The load of agricultural sources was apportioned equally over reaches based on the spatial linkage between land-use/land-cover (LULC) (Figure 4) and river systems.

The domestic source loads were allocated to the corresponding reaches through spatial correlations between residential districts and reaches.

The loads from large-scaled farming were allocated into the appropriate reach which were geographically closest to the farms based on longitude and latitude coordinates.

Therefore, the total contaminants discharged to the river and stream systems are calculated as the summation of all components.

(2) $$L_{j}=\sum_{i=1}^{m}E_{ij}A_{ij},$$

where $L_{j}$ is the discharge of contaminant j in the basin (kg); $E_{ij}$ is the export coefficient of the i th land-use type (kg/hm^2^) or the number of the ith poultry for contaminant j; $A_{i}$ is the area of the ith land-use type (hm^2^) or the discharge coefficient of the i th poultry (kg/yr).

### 2.4. Water Quality Response Models

#### 2.4.1. Determination of Stream Segmentation and Hydraulic Characteristics

**Figure 5:**
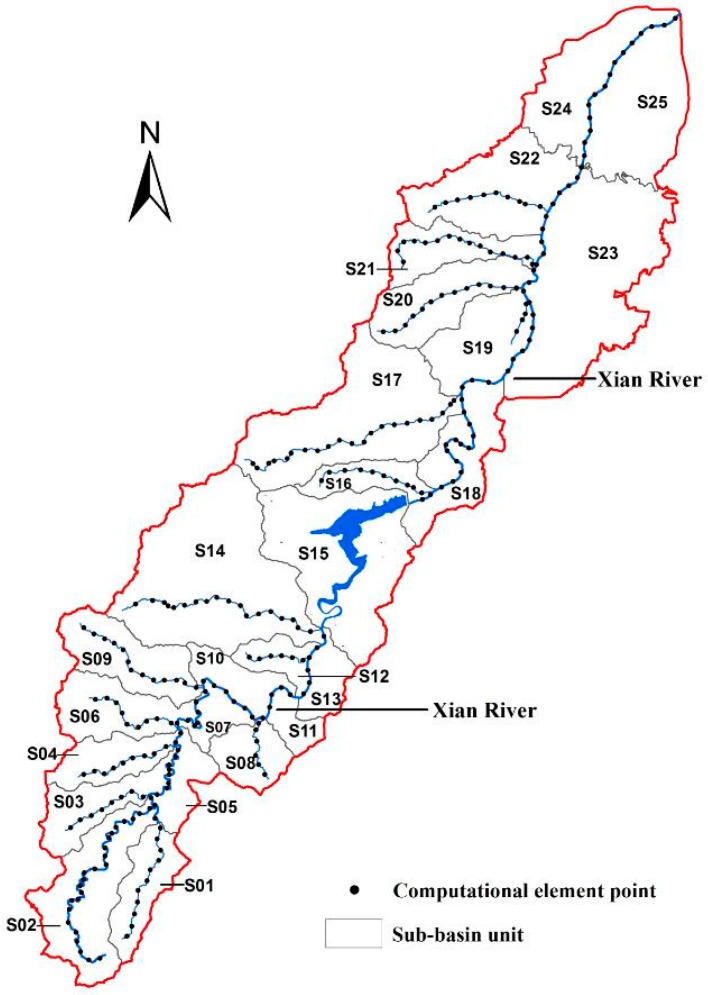
Sub-basin units and system elements number in the Xian-Jiang watershed.

For advantageous analysis of WEC, the watershed area was divided into 25 sub-basins (S01–S25). The hydrologic network was extracted from the digital elevation model (DEM) data, using the function of hydrologic analysis in ArcGIS 10.2 (version 10.2, ESRI Inc., Redlands, CA, USA), where there was one main stream (Xian River) and fourteen tributaries (Figure 5). Each reach was then subdivided into uniform computational elements for the model. These were 100 m in size, almost ten times more than the average calculation precision, and the reaches were divided into 1350 segmentations in total. The properties of the segmentations (length, width, sides slope, channel gradient, and elevation) were measured or estimated using ArcMap 10.2 (version 10.2, ESRI Inc., Redlands, CA, USA). The serial numbers of sub-basins and computational elements are displayed in Figure 5.

#### 2.4.2. Hydrodynamic Process

Every-segmentation in the reaches can be conceptualized as a trapezoidal channel with steady flow. For each channel, the nature of the relationship between flow and water depth was described by the Manning equation, expressed as Equation (3) [30]. To calculate the depth, flow rate, bottom width, riverbed slopes and side slope factors of the elements were required. Bottom width, riverbed slopes, and side slope factors were extracted from the DEM and vector data of the watershed. The flow rate in each element was calculated using the data from the above-mentioned hydrologic analysis. The flow of the 875th element located at the outlet of the reservoir was set based on the monthly average values of the discharge of the reservoir. According to previous studies on the Manning coefficient of roughness, the suggested n is generally in the range of 0.015–0.15, depending on the various types of channels [31,32]. In this paper, empirical n was set as 0.04 based on the properties of the river channels in the basin.

(3) $$H_{k}=\frac{{\left(Qn\right)}^{3/5}{\left(B_{0}+H_{k-1}\sqrt{{S_{S1}}^{2}+1}+H_{k-1}\sqrt{{S_{S2}}^{2}+1}\right)}^{2/5}}{S^{3/10}\left[B_{0}+0.5\left(S_{S1}+S_{S2}\right)H_{k-1}\right]},$$

where, H = depth of the river (m); Q = flow of the river (m^3^/s); n = Manning roughness coefficient; $B_{0}$ = bottom width of the river (m); S = riverbed slope (^o^); and $S_{S1}$, $S_{S2}$ represent both side slope factors, respectively. k = 1, 2, ⋯, i, i is the number of iterations. When the estimation error $\varepsilon_{\alpha }$ ($\varepsilon_{\alpha }=\left|\frac{H_{k+1}-H_{k}}{H_{k+1}}\right|\times 100\%$) < 0.001%, the iteration process stops.

Having obtained the water depth of each element, it was then substituted into Equation (4) to calculate the cross-sectional area of the channel (m^2^):

(4) $$A_{c}=\left(B_{0}+0.5\left(S_{S1}+S_{S2}\right)H\right)H,$$

Finally, the cross-sectional area $A_{C}$ (m^2^) was applied to Equation (5) to obtain the flow velocity U (m/s) of each element:

(5) $$U=\frac{Q}{A_{C}},$$

#### 2.4.3. Pollutant Degradation Process

In this paper, a one-dimensional water quality model was chosen to simulate pollutant degradation in the rivers. Based on water balance relationships, the fundamental equation for water quality simulation (Equation (6)) can be written as described in Reference [33]:

(6) $$\frac{\partial c}{\partial t}+u\frac{\partial c}{\partial x}=E\frac{\partial^{2}c}{\partial x^{2}}-k_{1}c,$$

The rapidly flowing rivers are dominant in mountain areas due to elevation differences that transverse velocity gradient which is much greater than vertical velocity gradient in rivers. Effects of longitudinal dispersion of contaminant are negligible compared to advection (the longitudinal dispersion coefficient *E* = 0) [34,35,36], then the model can be expressed as:

(7) $$\frac{\partial c}{\partial t}+u\frac{\partial c}{\partial x}=-k_{1}c,$$

where c is the concentration of the water quality (mg/L); u is the flow velocity (m/s) that can be calculated using the Manning equation; t is the time of river water flowing through from the headwater to somewhere (s); x is the distance that river water flows through in time t (m), x(t)=ut, and $k_{1}$ is the pollutant degradation coefficient (*d*^−1^).

For initial conditions of x(t)=0 and $c=c_{0}$, the accurate solution of Equation (7) can be obtained as follows:

(8) $$c\left[x\left(t\right)\right]=c_{0}exp\left[-k_{1}x\left(t\right)/u\right],$$

When the initial concentration (x=0, $c=c_{0}$) is known (the headwater referred to the water quality standard of water source protection area in China; the reservoir outlet referred to the actual monitoring concentrations), the concentration of a certain pollutant at x distance can be calculated using Equation (8).

#### 2.4.4. Matrix Cacluation Algorithms

**Figure 6:**
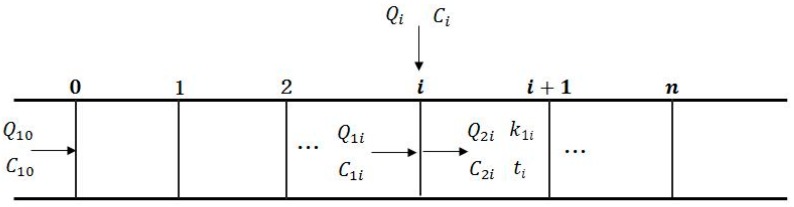
Schematic of conceptual model of one-dimensional steady-state river.

The process of little and nonpoint source input and degradation in the river is illustrated in Figure 6. In this figure, i represents the ith calculated element, i=1,2,⋯,n; $Q_{10}$, and $C_{10}$ represent the initial flow rate and concentrations from the headwater; $Q_{1i}$, and $C_{1i}$ represent the incoming flow rate and concentrations from the i−1th calculated element; $Q_{i}$, and $C_{i}$ represent the rainfall-runoff and pollutant concentrations draining into the ith calculated element; $Q_{2i}$, and $C_{2i}$ represent the outflow and concentrations of effluent discharging from the ith calculated element into the downstream; $k_{1i}$ represents the pollutant degradation coefficient between i and i+1; and $t_{i}$ represents the time consuming of flowing from i to i+1.

Based on solving the Streeter–Phelps equation, the changes in contamination concentration due to attenuation from i−1 to the i element can be described using Equation (9):

(9) $$C_{1i}=C_{2,i-1}\alpha_{i-1}\left(\alpha_{i-1}=e^{-k_{1,i-1}t_{i-1}}\right),$$

According to the principle of flow continuity, the balance of flow and concentrations for each node can be drawn using the following equations [33]:

(10) $$\{\begin{array}{c} Q_{2i}=Q_{1i}+Q_{i}\\ Q_{1i}=Q_{2,i-1}\\ C_{2i}Q_{2i}=C_{1i}Q_{1i}+C_{i}Q_{i} \end{array},$$

Then, Equations (9) and (10) can be derived using the following matrix equation:

(11) $$A\vec{C_{2}}=B\vec{C}+\vec{g},$$

where

(12) $$\{\begin{array}{c} \vec{C}={\left[\begin{array}{cccccc} C_{1} & C_{2} & \cdots & C_{i} & \cdots & C_{n} \end{array}\right]}^{T}\\ \vec{C_{2}}={\left[\begin{array}{cccccc} C_{21} & C_{22} & \cdots & C_{2i} & \cdots & C_{2n} \end{array}\right]}^{T}\\ \vec{g}={\left[\begin{array}{cccccc} g_{1} & 0 & \cdots & 0 & \cdots & 0 \end{array}\right]}^{T}\left(g_{1}=\alpha_{0}C_{20}\right) \end{array},$$

(13) $$A={\left[\begin{array}{cccccc} 1 & & & & & \\ -a_{1} & 1 & & & & \\ & -a_{2} & \ddots & & & \\ & & \ddots & \ddots & & \\ & & & \ddots & 1 & \\ & & & & -a_{n-1} & 1 \end{array}\right]}_{n\times n}\left(a_{n-1}=\frac{e^{-k_{1,n-1}t_{n-1}}Q_{1n}}{Q_{2n}}\right),$$

(14) $$B={\left[\begin{array}{cccccc} b_{1} & & & & & \\ & b_{2} & & & & \\ & & \ddots & & & \\ & & & \ddots & & \\ & & & & b_{n-1} & \\ & & & & & b_{n} \end{array}\right]}_{n\times n}\left(b_{n}=\frac{Q_{n}}{Q_{2n}}\right),$$

Having obtained the needed computational parameters, the little and nonpoint pollutant concentrations with $\vec{C}$ as the input, and the corresponding pollutant concentrations $\vec{C_{2}}$ as the output for each calculated element could be obtained. The computed pollutant concentrations of each element were applied to Equations (15) and (16) (below) to assess the water environmental capacity of each element.

#### 2.4.5. Water Environmental Capacity Calculation

To calculate the WEC of each element with limited data, a simple and proper computation model is needed. In this study, a section control method (that has been one of the most frequently used algorithms) was adopted that can be expressed as Equation (11) [37], where $q_{l}$ (average discharge flow from the lth outfall) was far less than Q (reach designed flow of element i). The simplified model can be expressed using Equation (12).

(15) $$W_{i}=Q_{i}\left(C_{Si}-C_{0i}\right)+Q_{i}C_{Si}\left(e^{k_{i}t_{i}}-1\right)+\sum_{l=1}^{n}q_{l}C_{si}+C_{si}\sum_{l=1}^{n}q_{l}\left(e^{k_{i}t_{i}}-1\right),$$

(16) $$W_{i}=Q_{i}\left(C_{Si}-C_{0i}\right)+Q_{i}C_{Si}\left(e^{k_{i}t_{i}}-1\right),$$

where $W_{i}$, $C_{Si}$, and $C_{0i}$ represent the water environmental capacity, the target concentration of the water quality, and the actual concentration of the pollutant of element i, respectively; $k_{i}$ is the degradation coefficient of the pollutant in element i, and $t_{i}$ is the time consuming of flowing through the element i.

Eventually, the contamination concentration and WEC of each calculated element i was simulated through the one-dimensional pollutant-water response model mentioned above. The calculation procedure of all models was programmed using MATLAB R2012b (version 8.0, The MathWorks, Natick, MA, USA).

## 3. Results and Discussion

### 3.1. Model Calibration and Verification

Water quality data (COD, NH_3_-N, and TP) of the monitoring section in the basin collected by the Zhejiang Province environmental monitoring station were used to calibrate and validate the water quality model at the beginning of the study. To minimize errors between the simulation results and observed data, the constants of COD, NH_3_-N, and TP degradation ($k_{1}$) were appropriately adjusted by trial and error, and were 0.1 day^−1^, 0.08 day^−1^, and 0.08 day^−1^, respectively [38,39]. The measured data inserted into the model for calibration and verification were monthly values (the mean of three water samples collecting in a month) from January to December 2015. Data from single months were used for calibration, and data from double months were used to verify the model. A comparison of the calibration results and the field measurements, in general, agreed well with the monitoring data, with the exception of a few values. For example, the simulated results of July and August deviated from the observed values most likely due to the delay of gathering water samples in a torrential downpour period. The root mean square error (RMSE) of COD, NH_3_-N, and TP between the simulated and measured values in the rest of the months of 2015 (excluding July and Augest) were 6.28, 0.39, and 0.08, respectively, and met the requirement of precision. Therefore, the models were acceptable to simulate the water quality and WEC under the condition of limited data. It should be noted that it does not fully validate a watershed model [18].

### 3.2. Contaminant Loadings Analyses

The pollutant quantity inlets into the river directly relates to the degree of contamination in the Xian-Jiang Basin. According to the calculation results, the total emissions into the river of COD, NH_3_-N, and TP in 2015 were 6719.68 *t*/a, 488.12 *t*/a, and 128.57 *t*/a for the Xian-Jiang Basin, respectively. Furthermore, the total contamination loads into the river in 2015 based on the type of pollution source were calculated (Table 1). COD discharge was due to agricultural nonpoint sources, formalization cultivation, domestic sewage, industrial enterprises above the designated size and wastewater treatment plant were 1188.78 *t*/a, 204.25 *t*/a, 4519.20 *t*/a, 65.68 *t*/a, and 741.77 *t*/a respectively, and accounted for 17.69%, 3.04%, 67.25%, 0.98%, and 11.04% of the total COD discharge in the watershed, respectively. Domestic sewage and agricultural nonpoint sources have been identified as the most significant sources of COD pollution, while industrial enterprises above the designated size and formalization cultivation were the least significant sources. The discharge and proportion of domestic sewage, agricultural nonpoint sources, wastewater treatment plants, formalization cultivation, and industrial enterprises above the designated size for NH_3_-N were 388.96 *t*/a (79.69%), >46.03 *t*/a (9.43%), >26.33 *t*/a (5.39%), >22.06 *t*/a (4.52%), and >4.74 *t*/a (0.97%). The domestic sewage (76.01 *t*/a) and agricultural nonpoint sources (24.86 *t*/a) were the two biggest contributors for TP in the watershed, which accounted for 59.02% and 19.34% of the aggregate, respectively. The discharge and proportion of wastewater treatment plant and formalization cultivation were 15.04 *t*/a (11.70%) and 12.35 *t*/a (9.61%), respectively.

### 3.3. The Spatio-Temporal Analyses of Water Quality

**Figure 7:**
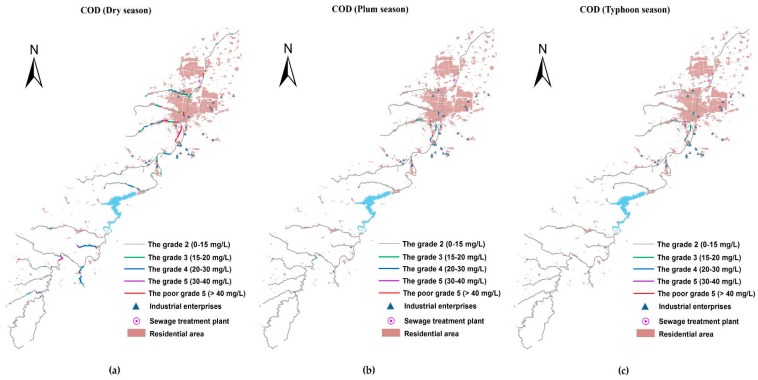
The space–time distribution of COD water quality standard for the Xian-Jiang Basin: (**a**) concentrations in the dry season; (**b**) concentrations in the plum rain season; and (**c**) concentrations in the typhoon season.

**Figure 8:**
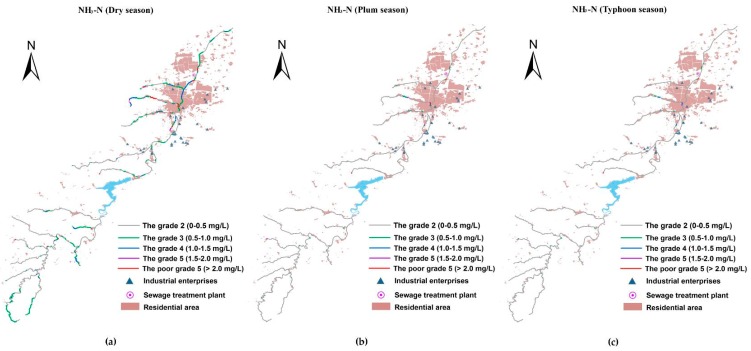
The space–time distribution of NH_3_-N water quality standard for the Xian-Jiang Basin: (**a**) concentrations in the dry season; (**b**) concentrations in the plum rain season; and (**c**) concentrations in the typhoon season.

**Figure 9:**
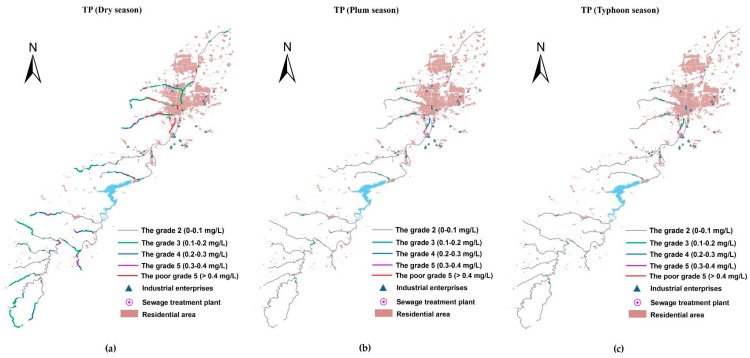
The space–time distribution of TP water quality standard for the Xian-Jiang Basin: (**a**) concentrations in the dry season; (**b**) concentrations in the plum rain season; and (**c**) concentrations in the typhoon season.

With the Manning formula-SP model simulation, the contaminant concentrations of the river system in the basin were obtained. Figure 7, Figure 8 and Figure 9 present the spatial distribution of water quality standards of COD, NH_3_-N, and TP in three different seasons, respectively. As shown, the water quality of the pollutants exhibited a seasonal variability, which in the dry season was demonstrably worse than in other seasons. The water quality during the plum rain season and typhoon season was in a comparable condition, and both were superior to the water quality objective in the majority of the reaches. This is because during the dry season, there is much less precipitation than in other seasons, and the main contaminative source is domestic sewage in the basin leading to a small water volume containing masses of pollutants. For the plum rain and typhoon seasons, the volumes were both large enough to digest the pollution loads. Although the plum rain season has more precipitation than the typhoon season, at the same time, runoff and drainage carry more nonpoint source pollutants into the river. The seriously polluted reaches that were Grade III or even worse have similar spatial distribution between plum and typhoon seasons. Especially, for COD, the number of the reaches in plum season were slightly more than in typhoon season in the upstream watershed of the reservoir. Regarding the Xi-Xi River in the middle area of the lower reaches, the water quality in plum season was worse than in typhoon season in the same reaches.

Selecting the most inferior dry season as an example, water quality in the upper reaches of the reservoir was generally better than in the lower reaches, where there is a water source protection area and a few factories and settlements. Among the three kinds of pollutants, COD had the best status upstream of the reservoir, the concentration of which basically met the Grade II standards, with the exception of the two tributaries near the reservoir. However, for NH_3_-N and TP, few tributaries in the upper reaches were Grade III or even worse, where a large number of paddy fields were distributed on both sides (compared with the LULC in 2015) carried plenty of nitrogen and phosphorous into the river. Furthermore, the water quality of TP was more deteriorated than NH_3_-N in the same reaches, partly due to the excessive use of phosphatic fertilizer in China, and partly because phosphorous is usually considered as a limiting nutrient for aquatic bioactivity and is largely removed through biodegradation or sedimentation [19]. The massive flow rate in this area prevented TP degradation and settlement. Therefore, the rice area along the river in the protection zone must be replaced by eco-friendly types. The concentrations of COD, NH_3_-N, and TP increased acutely in the middle area of the lower reaches where industry and resident cluster areas are located. A focus on the water quality in this area showed that the concentration of COD in the main river (Xian River) was much better than that of the tributaries due to water yield. This was especially the case for the Xi-Xi River where almost a whole section of the river was worse than Grade V, which was mainly attributed to industrial wastewater discharge from many machinery manufacturing plants around the river containing heavy concentrations of COD, besides the municipal loads. The concentration of NH_3_-N in this area wholly violated the water quality standard of Grade III including the main river. The top three heaviest polluted reaches of this area included outlet reaches where the river water flows into the Xian River and is the local center of the residential area, and the middle of the Bei-Xi River, as well as the upper reach of the Xi-Xi River where intensive machinery manufacturing plants are located. The situation of TP was similar to NH_3_-N, which corresponded to the same location as the most seriously polluted area. To summarize, a compelling spatial correlation existed between the density of pollutants and the distribution of plants and settlements.

### 3.4. The Temporal Variability of WEC in the Basin

**Figure 10:**
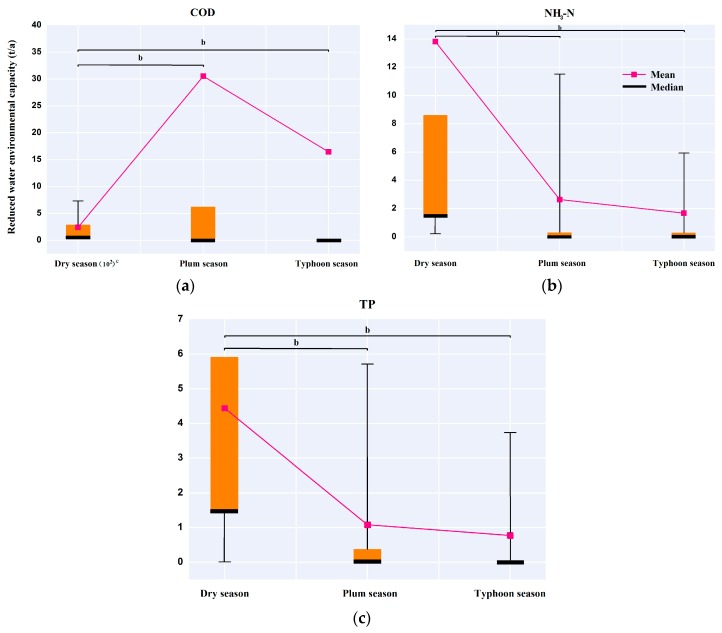
Difference in reduced WEC of pollutants among the three groups of seasons ^a^: (**a**) the reduced group of COD; (**b**) the reduced group of NH_3_-N; and (**c**) the reduced group of TP. ^a^ The figure shows the significant difference in reduced WEC among three groups of seasons with the Mann–Whitney U test. The top and bottom of each box represent the first and third quartiles with the median as a horizontal line inside. Whiskers are drawn as the 10th or 90th percentiles of the distribution; ^b^ Significant difference between hydrological seasons (*p* < 0.001 by the Mann–Whitney U test); ^c^ The unit of reduced WEC of COD in dry season: 10^2^ *t*/a.

The WEC of 24 sub-basins (excluding the S15 where the reservoir is located) as a group of subjects has been calculated for different hydrological seasons (dry season, plum rain season, and typhoon season), respectively. If the calculations of WEC were positive, it means the environmental capacity remained in surplus and could still accommodate the pollution load into the river with the premise of not violating the water quality standard. The positive values were replaced by zero indicating that no load needed to be cut; negative values indicated that the pollution load exceeded the environmental capacity and were replaced by its absolute values standing for the quantity that needed to be reduced. The independent-samples nonparametric statistical analyses with Mann–Whitney U tests were performed to assess group differences of the reduced WEC among the three hydrological seasons (Figure 10). A significance threshold of p was set as 0.001 and obtained from tables describing the critical values of test results using SPSS 18.0 software (version 18.0, IBM, Armonk, NY, USA).

As seen, the average reduced WEC of COD in the dry season was nearly eight and 15 times higher than in the plum rain and typhoon seasons, respectively (Mann–Whitney U-test, *p* < 0.001, one-sided, *n* = 24), indicating that there was a significant seasonal difference on COD gross reduction in the basin. The COD reduction had no obvious difference between the plum rain and typhoon seasons (Mann–Whitney U-test, *p* = 0.673, one-sided, *n* = 24). The dry season group also revealed a striking difference in reduced WEC of NH_3_-N with the plum rain and typhoon season groups (Mann–Whitney U-test, *p* < 0.001, one-sided, *n* = 24). Moreover, there was still no significant difference in a comparison of the plum rain season with the typhoon season (Mann–Whitney U-test, *p* = 0.572, one-sided, *n* = 24). As for TP, the distribution of the reduced WEC of the plum rain and typhoon season groups were far below that of the dry season group (Mann–Whitney U-test, *p* < 0.001, one-sided, *n* = 24). The average reduction of the dry season group (4.44 *t*/a) was 4.11 and 5.77 times that of the plum rain and typhoon season groups (plum season = 1.08 *t*/a; typhoon season = 0.77 *t*/a). No significant change was detected for the reduced WEC of TP between the plum rain and typhoon season groups (Mann–Whitney U-test, *p* = 0.419, one-sided, *n* = 24). Thus, the differences of reduced WEC in the basin for the three contaminants between the dry season and the other seasons were highly significant, suggesting that the reduced WEC exhibited a temporal variability that the pollution loads in the dry season were much more serious than other seasons in the study area.

### 3.5. The spatial Variability of WEC in Different Sub-Basins

**Figure 11:**
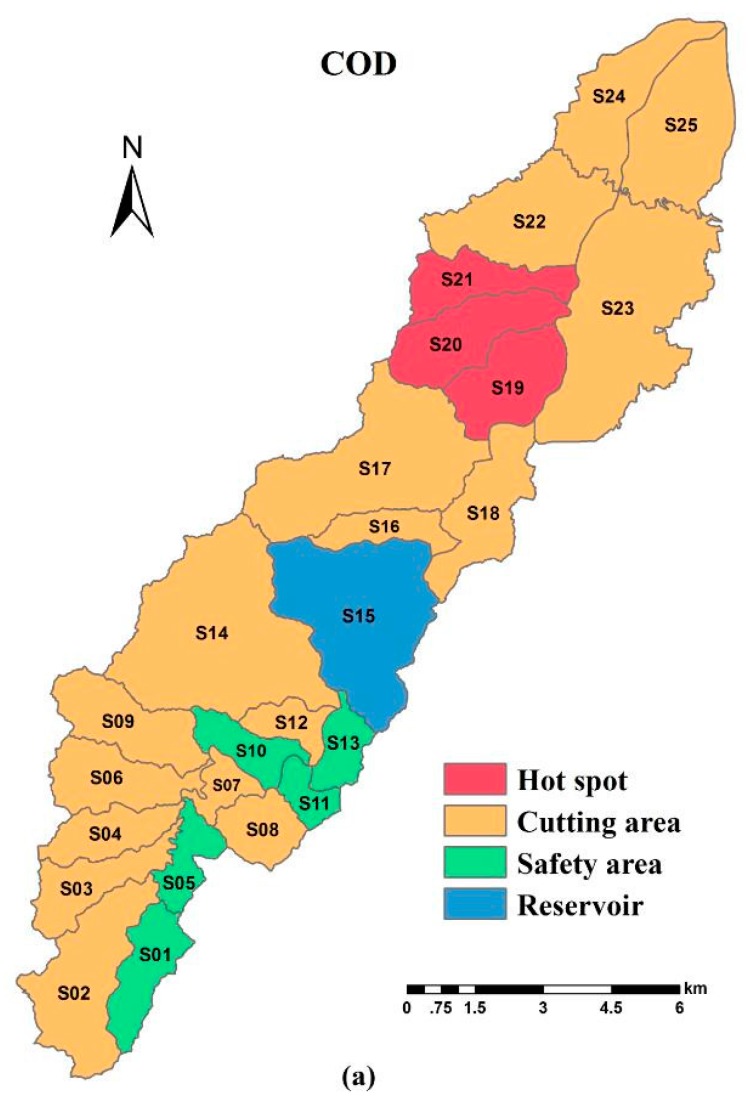

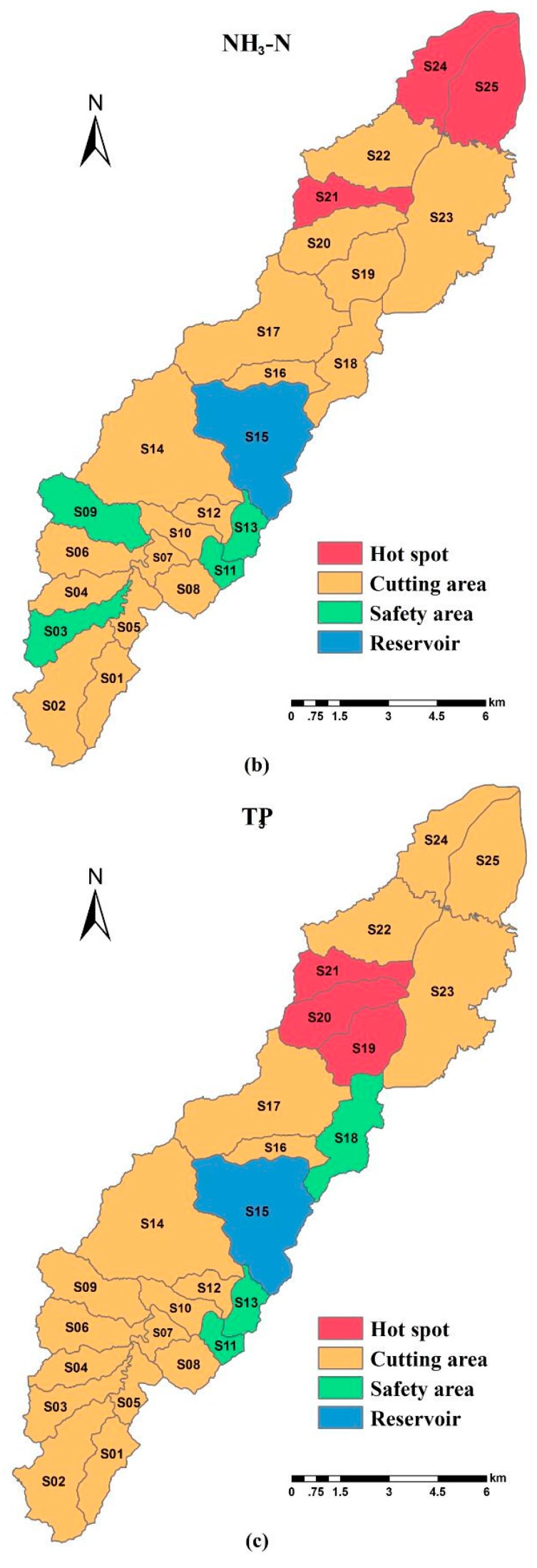
The spatial pattern of hot spots (the areas with the top three WEC reductions) and safety areas (the areas with no need to cut) of the Xian-Jiang sub-basins: (**a**) the spatial pattern of cutting areas for COD ; (**b**) the spatial pattern of cutting areas for NH_3_-N; and (**c**) the spatial pattern of cutting areas for TP.

The most polluted dry season was selected as the study period. The quantity of reduced WEC per unit area of contaminants in the 24 sub-basins was calculated (Table 2) and the descriptive statistics were described as per Table 3. The spatial pattern of hot spots and safety areas within the basin was shown in Figure 11 (Hot spots represent the areas with the top three WEC reductions; Cutting area represent the area needed to be reduced; Safety area represent the area with no need to cut). The distribution pattern of hot spots for COD and TP were exactly the same (S19, S20 and S21) and were the industrial and populated clusters, while they were mainly distributed in populated areas (S24 and S25) for NH_3_-N. These were the key regions of environmental management. Nearly all safety areas were located in upstream watershed of the reservoir where the water source conservation area is located. The average reduction of 24 sub-basins for the COD, NH_3_-N, and TP pollutants was 24.07 *t*/a, 1.12 *t*/a, and 0.73 *t*/a, respectively. Among them, the largest reduction for COD was in S19 with up to 132.95 *t*/km^2^·a^−1^; however, there were five sub-basins with a minimum of 0.00 *t*/km^2^·a^−1^, including S01, S05, S10, S11, and S13, indicating that there was still room to admit pollution loads in these regions. The most and least reduced regions for NH_3_-N were S21 (9.69 *t*/km^2^·a^−1^) and elements of S03, S09, S11, and S13 (0.00 *t*/km^2^·a^−1^), presenting a wide range of differences among the elements. Furthermore, S19 and S21 were the areas with the most TP reductions (2.07 *t*/km^2^·a^−1^) and the areas of S11, S13, and S18 still had a remaining environmental capacity (0.00 *t*/km^2^·a^−1^). As for statistical parameters of variation, the standard deviation of the reduced WEC for COD, NH_3_-N, and TP in the 24 sub-basins were 37.07 *t*/km^2^·a^−1^, 2.28 *t*/km^2^·a^−1^ and 0.61 *t*/km^2^·a^−1^, respectively, and the variation coefficient of the contaminants reached 154.01%, 203.57%, and 141.86%, respectively, the variability of which belonged to high levels [40]. Thus, the reduced WEC dramatically exhibited a spatial difference between all divisions of the basin during the dry season.

## 4. Conclusions

In 2015, COD, NH_3_-N, and TP discharge from diversified emission sources into the Xian-Jiang Basin were 6719.68 *t*/a, 488.12 *t*/a, and 128.57 *t*/a, respectively. Domestic sewage was identified as the primary source of all the contaminants, whereas industrial enterprises above the designated size were the least polluting sources for COD and NH_3_-N, with formalization cultivation for TP. According to the survey data, abundant domestic wastewater, especially for the villages, was not collected and accessed by the wastewater treatment plant through sewage conduits and was replaced by discharging into the river directly. Therefore, the increased acceptance rate and enhanced treatment capacity of domestic sewage has become very important and essential.

The modeling results indicated that the pollutant concentrations of the water system in the dry season were demonstrably worse than in the plum rain and typhoon seasons. The water qualities upstream of the reservoir, as a whole, were generally better than in the lower reaches where the concentrations basically met the Grade II standards. Furthermore, the middle part of the lower reaches was the most polluted waterbody as it was surrounded by industrial and residential cluster areas. The distribution of water quality standard exhibited a compelling correlation with the location of industrial enterprises, residential areas, as well as land-use types.

The WEC that needed to be eliminated for the basin to meet its water quality protective goal had a significant seasonal difference: the dry season ≫ the plum season > the typhoon season. Furthermore, there was also an obvious spatial difference in the reduced WEC between all sub-basins of the watershed. This finding implied that the current one-size-fits-all WEC cutting strategy adopted by environmental protection departments should be changed. Instead, WEC reduction should take spatio-temporal variation into account in the basin. If the reduced WEC during dry season can satisfy the required surface water quality standards, the probability of pollutant accidents is minimized annually where reducing the heavily polluted areas preferentially can help policy-makers save significant costs.

Although the finer the calculated elements, the higher the accuracy of water quality and WEC calculation becomes, this also increases the computation difficulty. Therefore, a balance point between the calculated elements scale and computation load requires further investigation. In addition, the model still has some deficiencies and room for improvement, such as different pollutant degradation coefficients that can be used considering the spatial heterogeneity of reaches, and increasing the monitoring sites for better calibration and verification. The water quality and environmental capacity simulation method discussed in this study can provide a good reference for other basins, especially for mountain river systems where long-term monitoring data are insufficient and confidential in China.

## Figures and Tables

**Table 1:** The contamination loads and proportion of pollution sources into the basin.

Pollution Sources	COD		NH_3_-N		TP	
	(*t*/a)	(%)	(*t*/a)	(%)	(*t*/a)	(%)
Agricultural Non-point source	1188.78	17.69	46.03	9.43	24.86	19.34
Formalization cultivation	204.25	3.04	22.06	4.52	12.35	9.61
Domestic sewage	4519.20	67.25	388.96	79.69	76.01	59.02
Industrial enterprises	65.68	0.98	4.74	0.97	0.31	0.33
Wastewater treatment plant	741.77	11.04	26.33	5.39	15.04	11.70
Total	6719.68	100.00	488.12	100.00	128.57	100.00

**Table 2:** Estimated WEC for the three contaminants in the sub-basins of the Xian-Jiang watershed during the dry season.

Sub-Basin ^1^	S01	S02	S03	S04	S05	S06	S07	S08	S09	S10	S11	S12
**Area**	7.61	16.33	8.04	6.79	4.77	9.64	3.47	6.01	10.84	5.27	3.12	4.00
**COD ^2^**	0.00	0.23	6.05	8.28	0.00	1.41	50.46	46.58	1.19	0.00	0.00	32.21
**NH_3_-N ^2^**	0.42	0.01	0.00	0.12	0.05	0.07	1.07	1.23	0.00	0.21	0.00	0.46
**TP ^2^**	0.17	0.20	0.16	0.08	0.07	0.06	0.93	0.56	0.07	0.16	0.00	0.36
**Sub-Basin**	**S13**	**S14**	**S16**	**S17**	**S18**	**S19**	**S20**	**S21**	**S22**	**S23**	**S24**	**S25**
**Area**	4.42	34.55	5.20	27.50	11.60	10.83	11.59	8.21	16.95	33.28	13.11	17.69
**COD ^2^**	0.00	0.25	10.37	4.07	0.99	132.95	109.50	79.79	7.12	3.49	41.40	41.40
**NH_3_-N ^2^**	0.00	0.12	0.08	0.02	0.02	0.79	0.92	9.69	0.20	1.58	4.94	4.94
**TP ^2^**	0.00	0.33	0.40	0.08	0.00	2.07	1.55	2.07	0.09	0.04	0.45	0.45

^1^ The catchment of Xian-Jiang Basin was divided into 25 sub-divisions (S01–S25), the location and area of which were shown in Figure 4 and Table 1. Area: km^2^; ^2^ The quantity of reduced WEC per unit area in 24 sub-basins. Area: km^2^. WEC: *t*/km^2^·a^−1^.

**Table 3:** Descriptive statistics of reduced WEC of contaminants in 24 sub-basins during the dry season.

Contaminants	N	Range ^1^	Min ^1^	Max ^1^	Mean ^1^	Median ^1^	SD	CV/%
**COD**	24	132.95	0.00	132.95	24.07	5.06	37.07	154.01
**NH_3_-N**	24	9.69	0.00	9.69	1.12	0.16	2.28	203.57
**TP**	24	2.07	0.00	2.07	0.43	0.17	0.61	141.86

^1^ The quantity of reduced WEC per unit area in 24 sub-basins. Area: km^2^. WEC: *t*/km^2^·a^−^^1^.

## References

[1] Miao M. (2009). Model and Enlightenment of economic development in coastal areas of China and Abroad. Econ. Rev..

[2] Singh R.B., Hales S., De Wet N., Raj R., Hearnden M., Weinstein P. (2001). The influence of climate variation and change on diarrheal disease in the Pacific Islands. Environ. Health Perspect..

[3] State Environmental Protection Administration of the China (SEPA) (2002). Environmental Quality Standards for Surface Water (GB3838-2002).

[4] Zhang R.B., Qian X., Zhu W.T., Gao H.L., Hu W., Wang J.H. (2014). Simulation and Evaluation of Pollution Load Reduction Scenarios for Water Environmental Management: A Case Study of Inflow River of Taihu Lake, China. Int. J. Environ. Res. Public Health.

[5] Liu Y., Zou R., Riverson J., Yang P., Guo H. (2011). Guided adaptive optimal decision making approach for uncertainty based watershed scale load reduction. Water Res..

[6] Liu R.M., Sun C.C., Han Z.X., Huang Q., Chen Y.X., Gao S.H., Shen Z.Y. (2012). Water environmental capacity calculation based on uncertainty analysis: A case study in the Baixi watershed area, China. Procedia Environ. Sci..

[7] Zhang Y.L., Liu P.Z. (1991). Comprehensive Manual of Water Environmental Capacity.

[8] Massachusetts Department of Environmental Protection TMDLs—Another Step to Cleaner Waters. http://www.mass.gov/eea/agencies/massdep/water/watersheds/tmdls-another-step-to-cleaner-waters.html.

[9] Li Y.X., Qiu R.Z., Yang Z.F., Li C.H., Yu J.S. (2010). Parameter determination to calculate water environmental capacity in Zhangweinan Canal Sub-basin in China. J. Environ. Sci..

[10] Royer T.C. (1979). On the effect of precipitation and runoff on coastal circulation in the Gulf of Alaska. J. Phys. Oceanogr..

[11] Hoyos C.D., Webster P.J. (2007). The role of intraseasonal variability in the nature of Asian monsoon precipitation. J. Clim..

[12] Ning S.K., Chang N.B., Yang L., Chen H.W., Hsu H.Y. (2001). Assessing pollution prevention program by QUAL2E simulation analysis for the Kao-Ping River Basin, Taiwan. J. Environ. Manag..

[13] Cooter W.S. (2004). Clean Water Act assessment processes in relation to changing US Environmental Protection Agency management strategies. Environ. Sci. Technol..

[14] Dobbins W.E. (1964). BOD and oxygen relationship in streams. J. Sanit. Eng. Div..

[15] Hosseini N., Chun K.P., Lindenschmidt K.E. (2016). Quantifying spatial changes in the structure of water quality constituents in a large prairie river within two frameworks of a water quality model. Water.

[16] Park S.S., Lee Y.S. (1996). A multiconstituent moving segment model for water quality predictions in steep and shallow streams. Ecol. Model..

[17] Zhang R., Qian X., Yuan X., Ye R., Xia B., Wang Y. (2012). Simulation of water environmental capacity and pollution load reduction using QUAL2K for water environmental management. Int. J. Environ. Res. Public Health.

[18] Lindenschmidt K.E. (2006). The effect of complexity on parameter sensitivity and model uncertainty in river water quality modelling. Ecol. Model..

[19] Fang X., Zhang J., Chen Y., Xu X. (2008). QUAL2K model used in the water quality assessment of Qiantang River, China. Water Environ. Res..

[20] Fan C., Ko C.H., Wang W.S. (2009). An innovative modeling approach using Qual2K and HEC-RAS integration to assess the impact of tidal effect on River Water quality simulation. J. Environ. Manag..

[21] Ningbo Municipal Water Conservancy Bureau Water Information Annual Report of Ningbo in 2016. http://www.nbwater.gov.cn/News_view.aspx?ContentId=26058&CategoryId=15.

[22] Gu C., Hu L., Zhang X., Wang X., Guo J. (2011). Climate change and urbanization in the Yangtze River Delta. Habitat Int..

[23] Zhou Y., Fu J.S., Zhuang G., Levy J.I. (2010). Risk-based prioritization among air pollution control strategies in the Yangtze River Delta, China. Environ. Health Perspect..

[24] Hsu K., Gupta H.V., Sorooshian S. (1995). Artificial neural network modeling of the rainfall-runoff process. Water Resour. Res..

[25] Luo W.S., Song X.Y. (2010). Engineering Hydrology and Water Conservancy Calculation.

[26] Johnes P.J. (1996). Evaluation and management of the impact of land use change on the nitrogen and phosphorus load delivered to surface waters: The export coefficient modelling approach. J. Hydrol..

[27] Wu Y.M., Li W., Yu Y.W., Li R.B., Long B.B., Shi J.Y. (2012). Non-point Source Pollution Loadings in Xitiaoxi. Watershed of Anji County, Zhejiang Province, China. J. Agro-Environ. Sci..

[28] Qian X.H., Xu J.M., Si J.C., Liu X.M. (2002). Comprehensive survey and evaluation of agricultural nonpoint source pollution in Hang-Jia-Hu water-net plain. J. Zhejiang Univ..

[29] Yan L.Z., Shi M.J., Wang L. (2010). Review of agricultural non-point pollution in Taihu Lake and Taihu Basin. China Popul. Resour. Environ..

[30] Fetter C.W. (2000). Applied Hydrogeology.

[31] Gordon N.D., McMahon T.A., Finlayson B.L., Gippel C.J., Nathan R.J. (2004). Stream Hydrology: An Introduction for Ecologists.

[32] Ding Y., Jia Y., Wang S.S. (2004). Identification of Manning’s roughness coefficients in shallow water flows. J. Hydraul. Eng..

[33] Fu G.W. (1987). Mathematical Model of River Water Quality and Its Simulation.

[34] White J.D., Dracup J.A. (1977). Water quality modeling of a high mountain stream. Water Pollut. Control Fed..

[35] Launay M., Le Coz J., Camenen B., Walter C., Angot H., Dramais G., Faure J.B., Coquery M. (2015). Calibrating pollutant dispersion in 1-D hydraulic models of river networks. J. Hydro-Environ. Res..

[36] Aksoy H., Kavvas M.L. (2005). A review of hillslope and watershed scale erosion and sediment transport models. Catena.

[37] Li X.L., Yao J., Zhang J., Zhao S. (2011). A comparative study of assimilative capacity for different pollution control modes in a river. Environ. Sci. Technol..

[38] Černý V. (1985). Thermodynamical approach to the traveling salesman problem: An efficient simulation algoritm. J. Optim. Theory Appl..

[39] Putnam H. (1965). Trial and error predicates and the solution to a problem of Mostowski. J. Symb. Log..

[40] Goovaerts P. (2001). Geostatistical modelling of uncertainty in soil science. Geoderma.

